# A Range-Based Algorithm for Autonomous Navigation of an Aerial Drone to Approach and Follow a Herd of Cattle

**DOI:** 10.3390/s21217218

**Published:** 2021-10-29

**Authors:** Manaram Gnanasekera, Jay Katupitiya, Andrey V. Savkin, A.H.T. Eranga De Silva

**Affiliations:** School of Mechanical and Manufacturing Engineering, The University of New South Wales, Sydney, NSW 2052, Australia; m.gnanasekera@unsw.edu.au (M.G.); j.katupitiya@unsw.edu.au (J.K.); eranga.desilva@unsw.edu.au (A.H.T.E.D.S.)

**Keywords:** aerial drones, unmanned aerial vehicles, UAVs, autonomous navigation, herding, animal farming, sliding mode control, range only measurements, range-based guidance

## Abstract

This paper proposes an algorithm that will allow an autonomous aerial drone to approach and follow a steady or moving herd of cattle using only range measurements. The algorithm is also insensitive to the complexity of the herd’s movement and the measurement noise. Once arrived at the herd of cattle, the aerial drone can follow it to a desired destination. The primary motivation for the development of this algorithm is to use simple, inexpensive and robust sensing hence range sensors. The algorithm does not depend on the accuracy of the range measurements, rather the rate of change of range measurements. The proposed method is based on sliding mode control which provides robustness. A mathematical analysis, simulations and experimental results with a real aerial drone are presented to demonstrate the effectiveness of the proposed method.

## 1. Introduction

Animal husbandry has been a social practice ever since the Neolithic Revolution. However, the techniques and technologies used in animal farming have evolved with time. As a result, modern-day farmers are progressively taking up technology in many ways to succeed in their farming endeavours. Automated feeding systems [1,2,3], smart farming techniques [4,5,6], robots used in farming [7,8,9], and intelligent farming technologies [10,11,12] can be highlighted as examples out of the vast range available in the literature.

Keeping track of farm animals is an essential task for a farmer, especially for the safety of animals and others. Sheepdogs and shepherds used in olden days have been replaced by technology in today’s farming. An extensive amount of literature can be found on animal tracking such as the work of [13,14,15,16] based on GPS. Specifically, most modern-day cattle tracking is done by attaching a GPS collar to the neck of the animal, as presented in [17,18]. Apart from the use of GPS collars, some other studies have used leg sensors [19] and ear sensors [18] for behaviour monitoring and location monitoring. Some researchers used different approaches, such as Wi-Fi [19], solar-powered sensors [20], and VHF collars [21] for livestock localization. Other than technological monitoring, the study conducted by [22] presents a mobile robot used for flock controlling. Method proposed in [23] uses a distributed game theoretic approach [24] to guarantee the safety of the herd from a potential predation.

The study carried out by [25] presents a method for bird herding using an aerial drone, also known as an Unmanned Aerial Vehicle (UAV), based on a waypoint algorithm. Similarly, some studies present how drone-based bird herding could be applied around airports [26,27] using a *n*-wavefront algorithm.

We draw our attention toward manoeuvring an aerial drone to an animal herd some distance away and to move along with the herd while maintaining a predefined altitude. Similar to military [28,29], surveying [30,31], cinematography [32,33], transportation [34,35] etc. sectors, agriculture [36,37] is one area where UAVs are used in large scales. Precision, easy deploy-ability, security, cost and flexibility are the key features behind the increased UAV usage [38].

This problem could be related to navigating an autonomous vehicle towards a static or dynamic target [39]. An extensive amount of literature could be found on ground vehicles moving towards targets that are developed based on various control laws. The study carried out by the authors of [40] have used a biologically inspired controller for vehicle guidance. The work presented in [41] proposed a control law which only uses angular information as inputs. As a result, the algorithm has become robust for sensing uncertainty. There were also guidance laws made to navigate wheeled robots towards static or moving targets based on range information [42,43]. It should be noted that sliding mode controllers based navigation laws are the backbone of most of these approaches, as can be found in [42,43,44,45] due to various advantages as described in [46]. In the case of aerial drones following ground targets, the work carried out is comparatively moderate. The work conducted by [47] uses sequential decision processing to track a mobile target. A Partially Observable Markov Decision Process (POMDP)-based method has been used by [48] to track ground targets. Some publications developed complex computer vision [49,50] and deep learning [51] algorithms for object tracking. The use of smarter, simpler, and practically implementable tracking methods-based control strategies are not widely available in the literature.

There are numerous methods presented in the literature to herd animals. The GPS collar methods stated above are not economically viable as GPS receivers with reasonable positioning accuracy are expensive. Moreover, GPS readings are generally inaccurate in remote areas unless some means of providing GPS corrections are made available. Secondly, the solutions which use LiDAR and vision cameras will suffer from limited range, limited field of view, poor visibility and occlusion. This type of sensors also have a very poor signal to noise ratio over long distances. Furthermore, they need to be pointed in the right direction for their readings to be useful. The directional information is not available at the start; hence this type of sensors cannot be used.

There are various methods used to navigate UAVs, including vision-based systems [52], GNSS-based systems [53] and on board short range sensors [54]. However, it is impossible to use vision and short range sensing for long range herding applications. Even though the satellite-based positioning systems such as GNSS could be used in long range applications, the positional inaccuracy at times could be hazardous and dangerous for UAV applications.

The proposed method uses simple range sensing, and the rate of change of range measurements is used to fly the drone towards the herd. The proposed algorithm is insensitive to the accuracy of range measurements. The algorithm only requires the rate of change of range. In practice, several transmitter/receiver modules should be used. One of the range sensing modules is placed on the aerial drone, and the other modules are attached to some of the cattle in the herd. The average range sensed by the drone can be taken as representative of the distance to the herd. In comparison to other sensing methods, range sensing is economical and robust.

Please note that range data does not give the directional information and hence the range alone does not indicate the location of the herd. Instead of a unique destination, a range sensor provides a large subset of possible destinations. Hence a search type algorithm must be developed in contrast to an algorithm that is deterministic such as an algorithm that pursues a known destination. Moreover, given that range data are less accurate, an algorithm that depends on the rate of change of range data is more suitable than an algorithm that depends on accurate range sensing. Sliding mode controllers are ideal candidates to operate in the presence of uncertainties. Hence the algorithm developed in this paper is based on sliding mode control methodology.

In this paper, we formulate the problem of drone navigation for following a ground-based herd, with an aerial receiving range-only measurements. The drone navigation law is based on sliding mode control. The distance from the drone to the herd will be taken as the range measurement. No bearing information is needed. The results shown in this work confirm the validity of the algorithm and its suitability to practical applications.

The following list of contributions are made to the existing knowledge base.

- The paper presents a control algorithm for a UAV to follow and intercept the location of a ground herd. To the best of the authors’ knowledge, the proposed work remains the only approach to address such an application.
- The control algorithm is based on range data. More importantly, the algorithm is robust against noisy data. The performance of the proposed method and a state of the art method was tested with noisy data in simulation. Our method has shown better performance than the state of the art method when compared.
- The UAV has the ability to intersect the herd’s location (finding the herd and moving along with it) and navigate along with it irrespective of the herd’s path complexity. We conducted multiple simulation and experiment tests in various complexity levels to confirm this point.

The rest of the paper content is organized as follows. Section 2 provides the problem statement and solution development, the main results and their mathematical analysis. Section 3 presents the descretized algorithm and the dynamic model. Computer simulation and experimental results are presented in Section 4 and Section 5 receptively. Section 6 concludes the paper.

## 2. Problem Statement and Solution Development

The problem to be solved is formulated as follows. An aerial drone is initially at a certain location (the farm station) of the farm, far removed from the grazing area of a herd of cattle. The grazing area is a substantially large area of land and the cattle as a herd may roam freely anywhere within the area. The resting place or the shed to which the cattle are herded for the night is located within this grazing area. Please note that this paper does not address the herding technologies that may be applied. The emphasis in this paper is locating the herd and then tracking the herd as it moves toward the grazing land as well as moving the herd back to the shed. Once the drone has intercepted the herd, the GPS coordinates of the drone may be used to log the movement of the herd and to verify the arrival of the herd at the shed.

The drone navigates by being at a constant height h>0 at all times. The location of the drone is given as (x(t),y(t)). The drone navigates in a ϕ(t) heading direction, which is measured anti-clock wise from the x−axis. Where −π<ϕ⩽π. Let the drone travel in a constant speed u>0 and an angular velocity $\dot{\phi }\left(t\right)$. Then, the translational motion could be introduced as (1).

(1) $$\begin{array}{cc} \dot{x}\left(t\right)= & u\cos(\phi (t));\\ \dot{y}\left(t\right)= & u\sin(\phi (t)). \end{array}$$

The limits of $\dot{\phi }\left(t\right)$ will always be between$-{\dot{\phi }}_{max}\leq \dot{\phi }\left(t\right)\leq {\dot{\phi }}_{max}$. Where ${\dot{\phi }}_{max}>0$.

The heard could be stationary or moving on the ground and the $\left(x_{H}\left(t\right),y_{H}\left(t\right)\right)$ becomes the coordinates of the herd. Let d(t) be the sole range information the drone has about the herd at any time *t*. Since, *h* height is perpendicular to the ground, we introduce l(t) as in (2). Let, e(t) be a minor error component that occurs due to signal noise. If *E* is a given constant, |e(t)|<E.

(2) $$\begin{array}{c} l\left(t\right)=\sqrt{\left(d^{2}\left(t\right)-h^{2}\right)}+e\left(t\right). \end{array}$$

**Definition** **1.** *The drone arrives at the herd’s location when l(T)⩽L, where T>0 and L∈R.*

Let g(t) be a given function, $g(t^{-})$ at *t* could be introduced as in (3).

(3) $$\begin{array}{c} g\left(t^{-}\right):=\lim_{\xi \to 0,\xi >0}g\left(t-\xi \right). \end{array}$$

We introduce the sliding mode control law as (4).

(4) $$\begin{array}{c} \dot{\phi }\left(0\right)={\dot{\phi }}_{max},\\ \dot{\phi }\left(t\right)=-\dot{\phi }\left(t^{-}\right)\mathrm{sgn}\left(\ddot{l}\left(t^{-}\right)\right). \end{array}$$

We introduce Assumption 1.

We assume that initially the drone is not too close to the herd’s location as in Assumption 1.

**Assumption** **1.** *The statement $l\left(0\right)>\frac{3u}{{\dot{\phi }}_{max}}$ holds all times.*

We introduce Lemma 1.

**Lemma** **1.** *Let the angle between the drone’s initial direction and the herd be $<\frac{\pi }{2}$ and with the aid of Assumption 1, it could be said that the control law (4) guides the drone towards the location of a herd which is stationary. Where $\left(x_{H}\left(t\right),y_{H}\left(t\right)\right)\equiv \left(X_{H},Y_{H}\right)$.*

**Proof** **of Lemma 1.** Let λ(t) be the angle between the drone’s heading ϕ(t) and the direction from the drone towards the herd. Then the following equations hold:

(5) $$\begin{array}{c} \dot{l}\left(t\right)=-u\cos\left(\lambda \left(t\right)\right); \end{array}$$

(6) $$\begin{array}{c} \dot{\lambda }\left(t\right)=-\dot{\phi }+\frac{u\sin(\lambda (t))}{d(t)}, \end{array}$$

see, e.g., [42]. Furthermore, there exist two circles of radius

(7) $$R:=\frac{u}{{\dot{\phi }}^{max}}$$

that is tangent to the drone’s initial heading ϕ(0). One of these circles has the property that it intersects the straight line segment connecting the drone’s initial position and the herd. It follows from (4) that the drone first moves along this circle until its heading ϕ(t) coincides with the direction towards the steady herd. Indeed, it follows from Assumption 1 that the herd is outside of this circle and the distance between the herd and any point of this circle is greater than $\frac{u}{{\dot{\phi }}^{max}}$. This and (6) imply that λ(t) is decreasing when the drone moves along this circle, hence, it follows from (5) that $\dot{l}\left(t\right)$ is decreasing, therefore, $\ddot{l}\left(t\right)<0$. Furthermore, it is obvious that if the heading ϕ(t) for some *t* coincides with the direction towards the steady herd, then the sliding mode control law (4) will keep the heading at the line connecting the drone with the steady herd which corresponds to the sliding surface $\ddot{l}\left(t\right)=0$. Hence, the drone will move towards the herd along this straight line and intercept it. This completes the proof of Lemma 1.   □

We introduce Assumptions 2 and 3 in order to consider the problem of moving herds $v_{H}\left(t\right):=\sqrt{{\dot{x}}_{H}{\left(t\right)}^{2}+{\dot{y}}_{H}{\left(t\right)}^{2}}$.

**Assumption** **2.** *Let $v_{max},k\in R$ such that $v_{H}\left(t\right)\leq v_{max}<u$ and $|\dot{u}\left(t\right)|\leq k$.*

**Assumption** **3.** *Let the condition ${\left(\frac{v_{H}}{u}\right)}^{2}+{\left(\frac{2k}{u{\dot{\phi }}^{max}}\right)}^{2}<1$ hold.*

Assumptions 2 and 3 guarantee that the speed and the acceleration of the herd is not too large compared to the drone’s speed and angular velocity. It is obvious that if the herd is moving too fast, interception is impossible. In practice, the drone’s speed and angular velocity are known a priori from drone’s specifications. The speed and the acceleration of the herd can be obtained from preliminary studies of herds of cattle using, for example, aerial video surveillance of herds.

**Lemma** **2.** *If all the above assumptions are sustained, the control law makes the drone navigate towards the moving herd and intercept if $D>\frac{u+v_{max}}{2{\dot{\phi }}^{max}}$.*

**Proof** **of Lemma 2.** Let λ(t) be the angle between the drone’s heading ϕ(t) and the direction from the drone towards the herd, β(t) be the angle between the herd’s heading and the direction from the herd towards the drone. Then the following equations hold:

(8) $$\begin{array}{c} \dot{l}\left(t\right)=-u\cos\left(\lambda \left(t\right)\right)+v_{H}\left(t\right)\cos\left(\beta \left(t\right)\right); \end{array}$$

(9) $$\begin{array}{c} \dot{\lambda }\left(t\right)=-\dot{\phi }+\frac{u\sin(\lambda (t))}{l(t)}-\frac{v_{H}\left(t\right)\sin\left(\beta \left(t\right)\right)}{l(t)}, \end{array}$$

see, e.g., [42]. Furthermore, as in the poof of Lemma 1, it follows from (4) that the drone first moves along the circle of radius (7) that is tangent to the initial drone’s heading ϕ(0) and intersects the straight line segment connecting the drone’s initial position and the herd’s initial position $\left(x_{H}\left(0\right),y_{H}\left(0\right)\right)$. Indeed, it follows from Assumption 1 that the herd is outside of this circle at time 0. Moreover, it follows from (4) and (9) that when the drone moves along this circle and $l\left(t\right)>\frac{u+v_{max}}{2{\dot{\phi }}^{max}}$ then $\dot{\lambda }\left(t\right)<-\frac{{\dot{\phi }}^{max}}{2}$. This and (9) imply that λ(t) is decreasing when the drone moves along this circle. Furthermore, it follows from (8) and Assumption 2 that $\ddot{l}\left(t\right)<0$ if $\dot{\lambda }\left(t\right)<-\frac{{\dot{\phi }}^{max}}{2}$. This, (8) and Assumption 3 imply that $\dot{l}\left(t\right)<-\xi <0$ if $l\left(t\right)>\frac{u+v_{max}}{2{\dot{\phi }}^{max}}$. Therefore, since *D* satisfy $D>\frac{u+v_{max}}{2{\dot{\phi }}^{max}}$, the proposed navigation law (4) is D−intercepting. This completes the proof of Lemma 2.   □

In the forthcoming sections, we tested the performance of the control law (4).

## 3. The Proposed Algorithm and the Dynamic Model

We introduce the following function where $\dot{l}\left(t\right)$ denotes the derivative of l(t).

(10) $$f\left(t\right)={\left(\dot{l}\left(t\right)-u\right)}^{2}$$

We introduce the following discretized version of the control law (4) in Algorithm 1. The sample time is given by δT and $\phi_{0}$ is a constant heading angle ($\phi_{0}\in R$).
**Algorithm 1** Calculating ϕ(δT) **Input:** f(δT), f((δ−1)T) **Output:** ϕ(δT)1:**if**f(δT)−f((δ−1)T)<0**then**2: ϕ(δT) ←  $\phi ((\delta -1)T)+\phi_{0}$3:**else**4: ϕ(δT) ←  $\phi ((\delta -1)T)-\phi_{0}$5:**end if**

We introduce the following PD controller in (11) to find the τ needed to control the heading angle. Kd,Kp∈R. ω(t) is the angular velocity, *I* is the inertia of the UAV and β is the Coulomb resistance.

(11) $$\tau =Kd\left(\dot{\phi }\left(\delta T\right)-\dot{\phi }\left(\left(\delta -1\right)T\right)\right)+Kp\left(\phi \left(\delta T\right)-\phi \left(\left(\delta -1\right)T\right)\right)$$

The motion model of the UAV can be introduced as (12).

(12) $$\begin{array}{ccc} \dot{x}\left(t\right) & = & u\cos(\phi (t))\\ \dot{y}\left(t\right) & = & u\sin(\phi (t))\\ \dot{\phi }\left(t\right) & = & \omega (t)\\ \dot{\omega }\left(t\right) & = & (-\beta /I)\omega (t)+\tau /I \end{array}$$

## 4. Simulation Results

We conducted Matlab-based simulations to test the performance of the proposed algorithm with both stationary and moving herds. The results of both cases are presented in this section. In all navigation (herd tracking) plots *x* and *y* coordinates are expressed in meters. It is important to note that u=5ms${}^{-1}$ in all simulations. In Section 4.1, the control algorithm has been tested with static and dynamic herds. There were five tests conducted with dynamic herds (Figure 2–6) and the complexities of the travel paths were varied to test the performance. Thereafter in Section 4.2, two tests have been conducted with noisy data. A state of the art method [55] was made to perform the same two tests and the results of the two methods are compared(the proposed algorithm and the state of the art method [55]).

### 4.1. Target Location Interception

**Figure 1:**
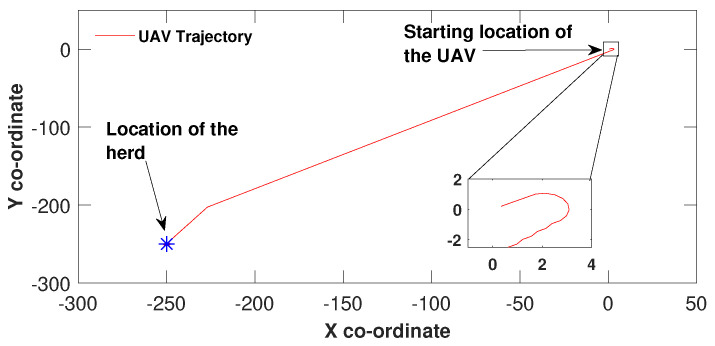
UAV travelling to the location of the stationary herd.

In Figure 1, the UAV’s task is to travel towards a herd stationed at the co-ordinates (−250, −250). Since the herd’s direction is different from the UAV’s initial heading, an initial heading change around 180${}^{\circ }$ could be observed. This heading change was made by the control law (4) of the sliding mode controller. Whenever the control law decides to use ϕ(t)<0 the control law is in sliding on the sliding manifold and when off the sliding surface ϕ(t)>0 is applied. In this simulation result, the transition from sliding surface to off the sliding surface and then back on to the sliding surface occurs around (−230, −198). This makes the controller change the heading of the drone to reach the herd’s location. The UAV has stopped at the herd’s location because this location corresponds to the origin of the sliding surface of the error driven sliding mode controller.

**Figure 2:**
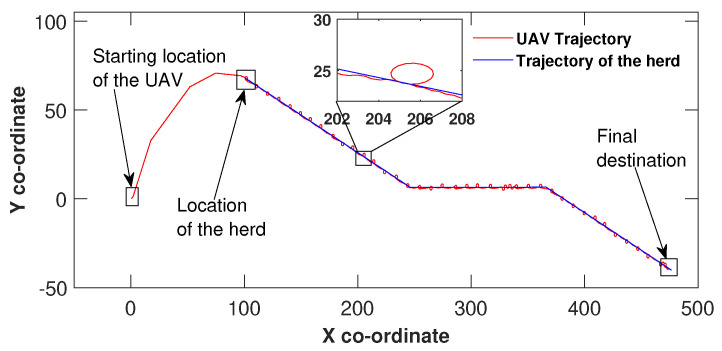
Herding on a piecewise linear path.

In Figure 2, the UAV reaches the herd’s location and tracks the herd as it moves towards the final destination. In this particular case, the control law (4) of the UAV maintains the control effort until the origin of the sliding surface is reached at co-ordinates (102,75), which is the herd’s location. From this point onwards, the UAV has moved along with the herd till the final destination. Please note that some circular movements of the UAV could be observed along the path taken by the herd (a location is zoomed). The main reason for this behaviour is that the UAV maintains a constant speed which is slightly greater than the speed at which the herd is moving. This causes the error states to shift off the siding mode, however, the sliding mode controller wrests control of the drone and brings back to the herd’s location.

**Figure 3:**
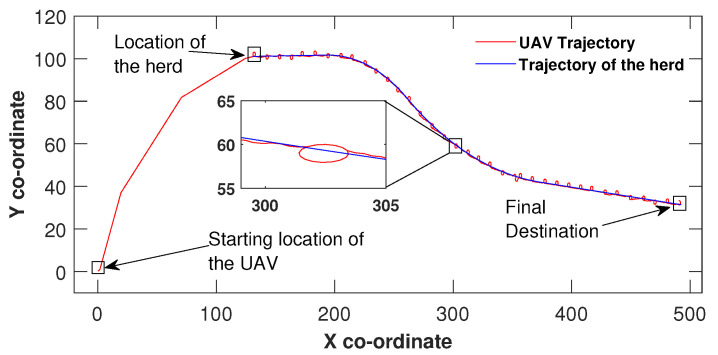
Herding on a curved path.

Similar to Figure 2, the UAV in Figure 3 maintained the control effort by making three heading changes before reaching the sliding surface origin around (149,100) and then manoeuvred hand in hand with the herd until the herd reached their final destination. The same UAV circulation could be identified due to the speed difference in this scenario as well. However, the only difference between Figure 2 and Figure 3 is the herd’s travel paths. In Figure 2, the path is made out of three linear segments and in Figure 3 the path is complex and consists some curved segments. In all three figures, the UAV found the herd’s initial location and has not missed the herd at any point while travelling towards the destination. These simulation results show the validity of the proposed algorithm.

**Figure 4:**
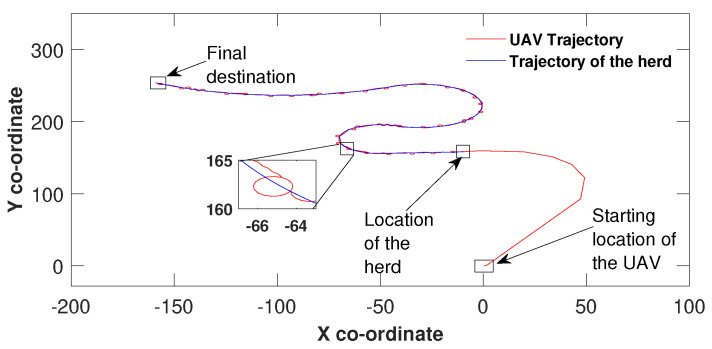
Herding on a complex path.

**Figure 5:**
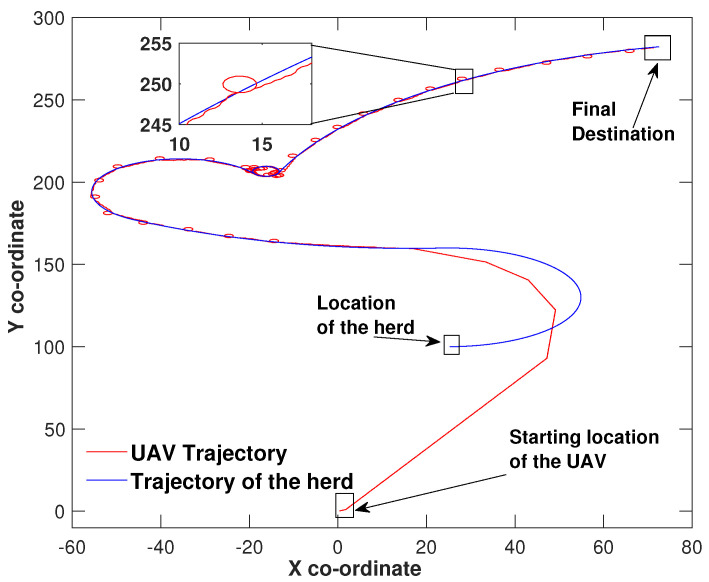
Herding on a second complex path.

**Figure 6:**
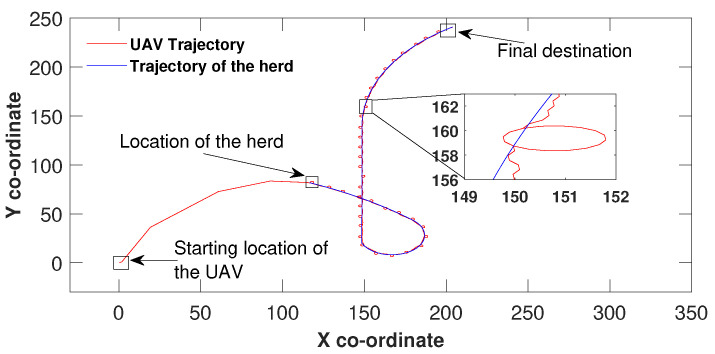
Herding on a third complex path.

To further investigate the robustness, the algorithm was applied to a herd which moves in complex paths (Figure 4, Figure 5 and Figure 6). Equation (4) has made the UAV in Figure 4 make six heading changes before approaching the herd. According to Figure 4 the UAV reached the target around co-ordinates (0,160). Similar to all other figures, the UAV travelled along with the target here onwards by being at the origin of the error space. In Figure 5, the UAV made five heading changes to reach the target around (10,160) which corresponds to the origin of the error space. The path towards the destination in this scenario is complex. However, the UAV successfully tracked the herd to the final destination despite the path complexity. Similar to Figure 5, Figure 6 shows the results of another complex simulation. The control algorithm of the UAV has made three heading changes before reaching the origin of the error space, at (125,75). Thereafter, similar to Figure 5 the UAV successfully tracked the herd towards the destination.

### 4.2. Performance under Noise

**Figure 7:**
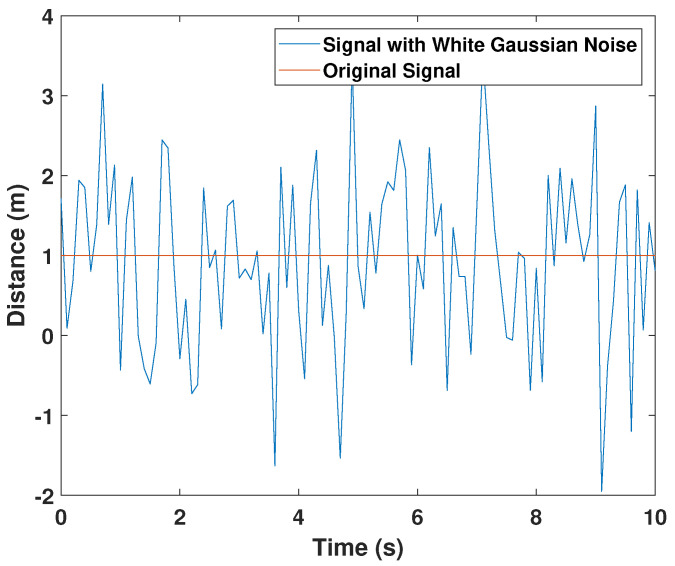
The White Gaussian Noise.

**Figure 8:**
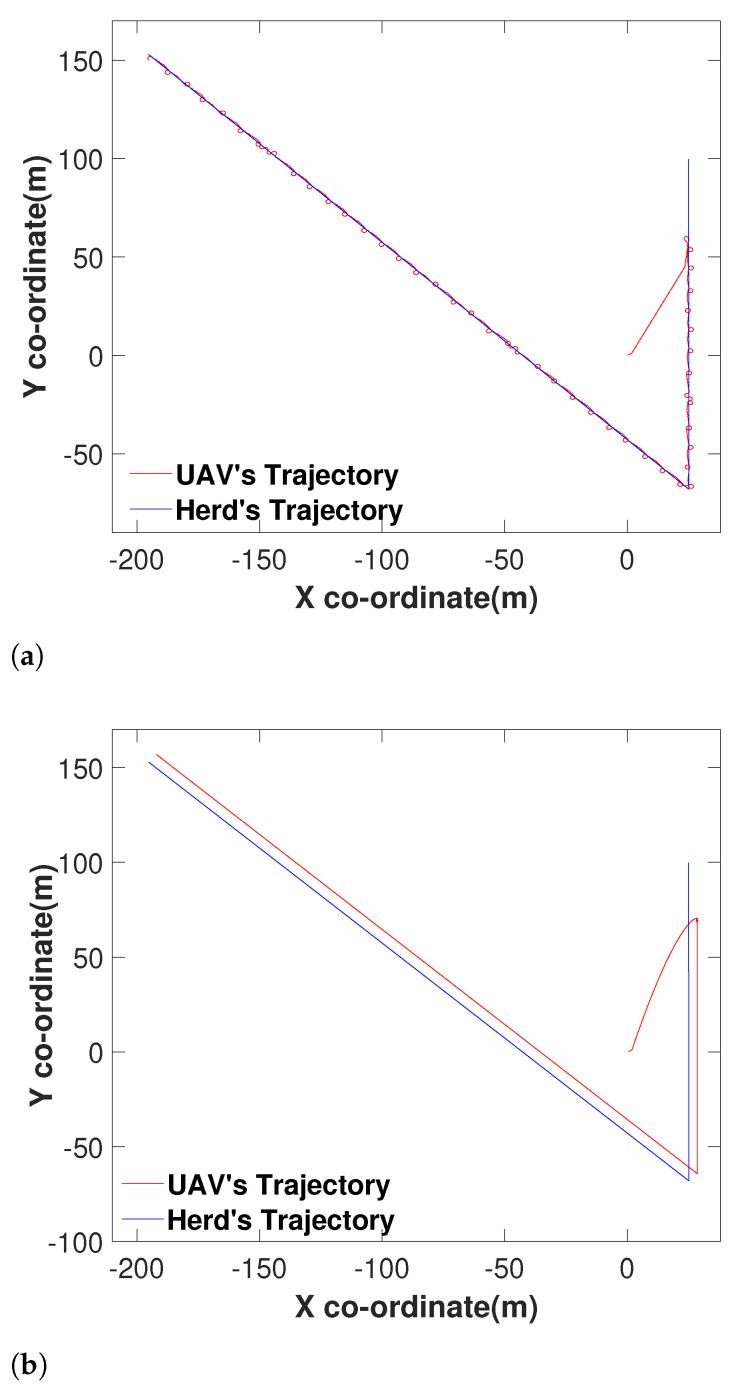
Comparison with a herd travelling in a linear trajectory: (**a**) The proposed method following the herd with noisy range signal data. (**b**) The performance of the method proposed in [55] when made to travel towards the same herd under the same circumstances.

To test the performance of the algorithm under noise, we introduced a white Gaussian noise with a 0.001 SNR. The noise signal is presented in Figure 7. We tested the performance of the UAV with a noisy range signal and made the UAV to navigate towards the target. The target travelled in a piecewise linear trajectory. To compare the result, we have also made the navigation algorithm introduced in [55] to follow the same target under noise. The obtained results are shown in Figure 8a. It is more than evident that the performance of the proposed method has not been disturbed by the noise according to Figure 8a. However, the method proposed in [55] has not been able to find the target location properly due to the effect of the noise (Figure 8b).

**Figure 9:**
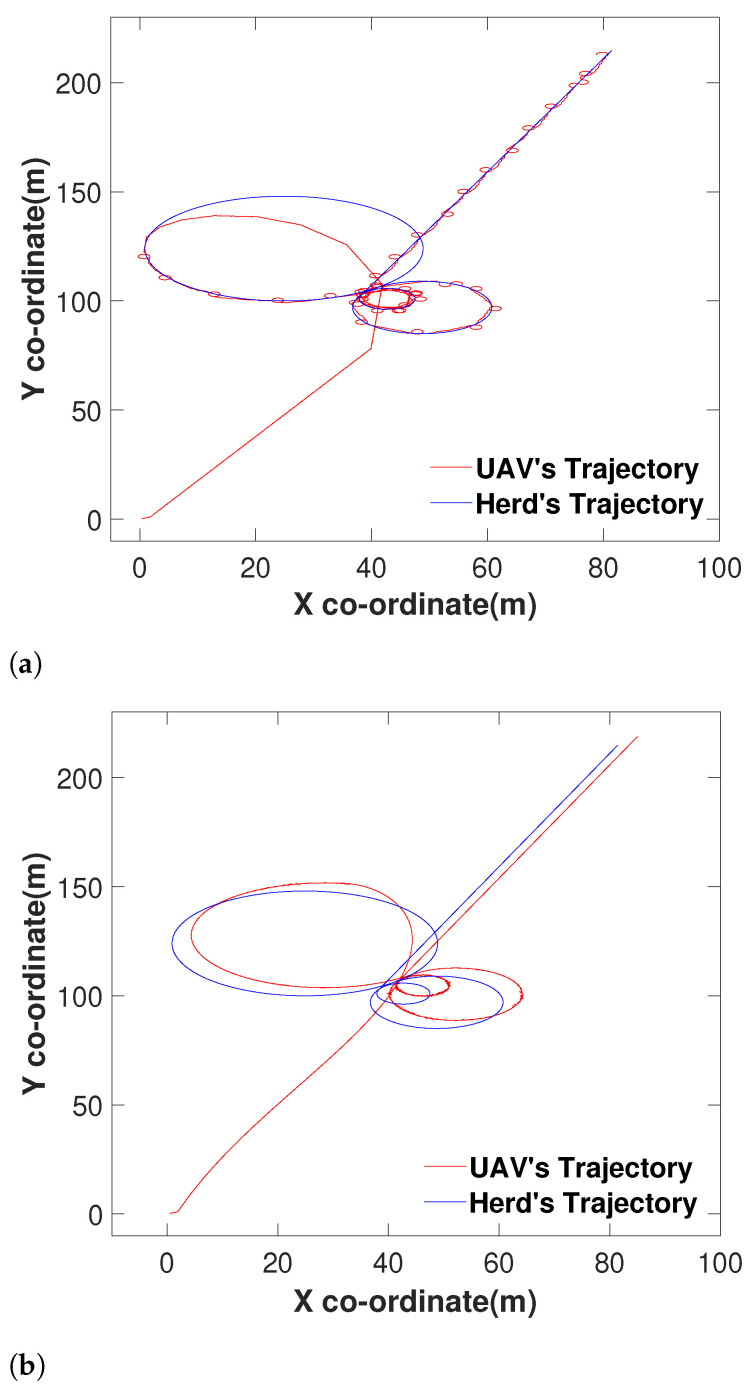
Comparison with a herd travelling in a complex trajectory: (**a**) The proposed method following the herd with noisy signal data. (**b**) The performance of the method proposed in [55] when made to travel towards the same herd under the same circumstances.

To make a proper conclusion, we also made both the algorithms travel towards a target travelling in a complex path (Figure 9). It is apparent that the proposed method performed well with the noisy range signal according to Figure 9a. On the other hand, the method proposed in [55] has not been successful similar to the previous example.

## 5. Experimental Results

**Figure 10:**
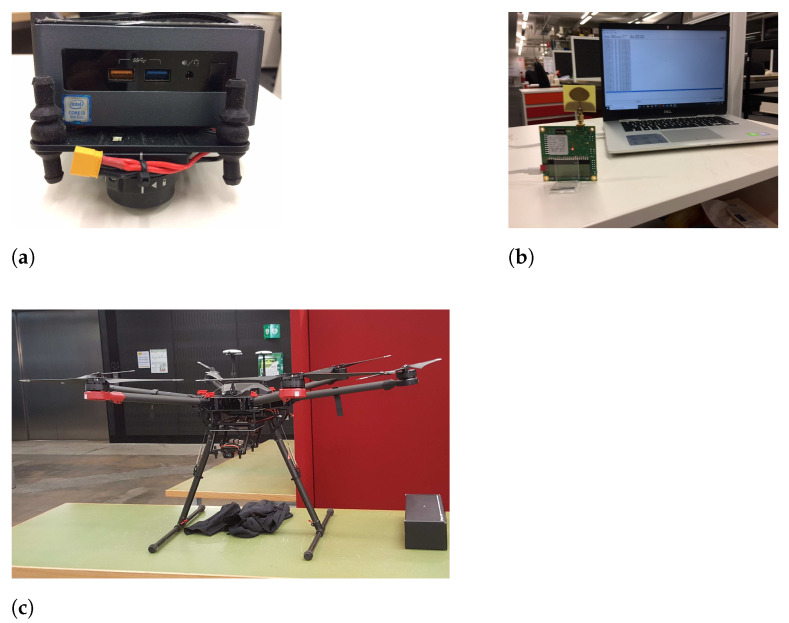
(**a**) Intel NUC Mini PC (**b**) Decawave Trek 1000 range sensor (**c**) Matrice 600 PRO drone used for the practical experiments.

**Figure 11:**
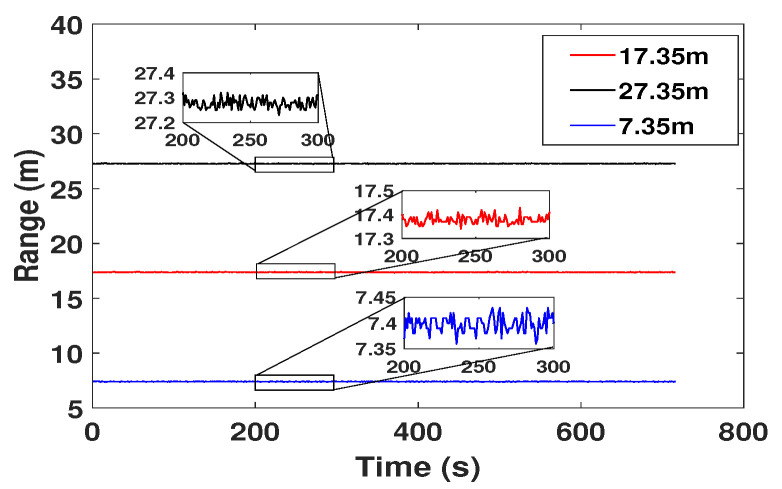
The plot shows the results of three different range measurements over a period of 12 min.

The proposed algorithm was experimentally tested using a DJI Matrice 600 Pro drone (Figure 10c). The drone was serially connected to an Intel NUC Mini PC (Figure 10a). We automated the drone with the aid of a DJI Onboard Software Development Kit Version 3.6 which was installed in the Mini Pc. The code was written in C++ for the experiments. A Deacawave Trek 1000 range sensor which could be deployed for measuring the range distances is shown in Figure 10b. Two of these range sensors are needed for the distance measurements (one should be fixed to the drone and the other should be connected to one of the cow’s in the herd) and the data transmission between the two sensors happens through Ultra Wide Band radio technology. Figure 11 shows the accuracy of three different range measurements over 720 s. The accuracy is within ±2cm.

**Figure 12:**
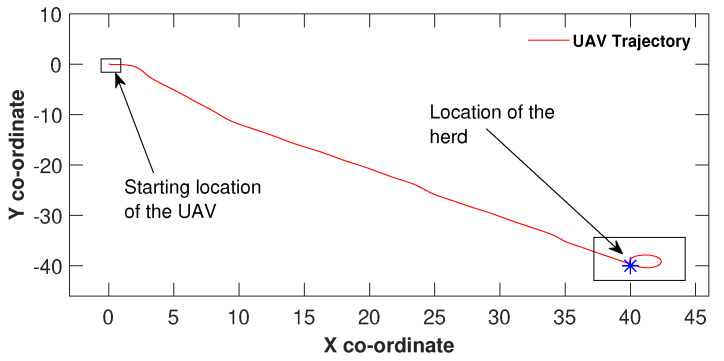
The drone navigating towards a static herd.

In the experiment, the drone’s speed was set to 0.21ms${}^{-1}$. In all navigation (herd tracking) plots *x* and *y* coordinates are expressed in meters. Unlike in the simulation scenario, the drone was subjected to disturbances such as wind effects during the experiment. From the starting co-ordinate till around (2.4, 0), the drone has had the initial heading. Thereafter, based on the range signal, the control law in Equation (4) has changed the heading direction towards the herd’s location and travelled directly towards the herd. However, it is obvious in Figure 12, the trajectory of the drone after the heading change has not been a straight line or a combination of straight lines. The leading cause of the drone’s wavered behaviour has been the resistance produced by the wind. The herd’s location corresponds to the origin of the error space. Furthermore, it also could be observed that the drone has circulated at the herd’s location. The reason for the circular movement of the drone is because the speed of the herd is 0, and the drone has got a speed which is higher than 0. The speed difference between the herd and the drone would give rise to the circulation. Apart from the circular movement at the herd’s location, Figure 12 justifies that the control law performs well when the herd is static.

**Figure 13:**
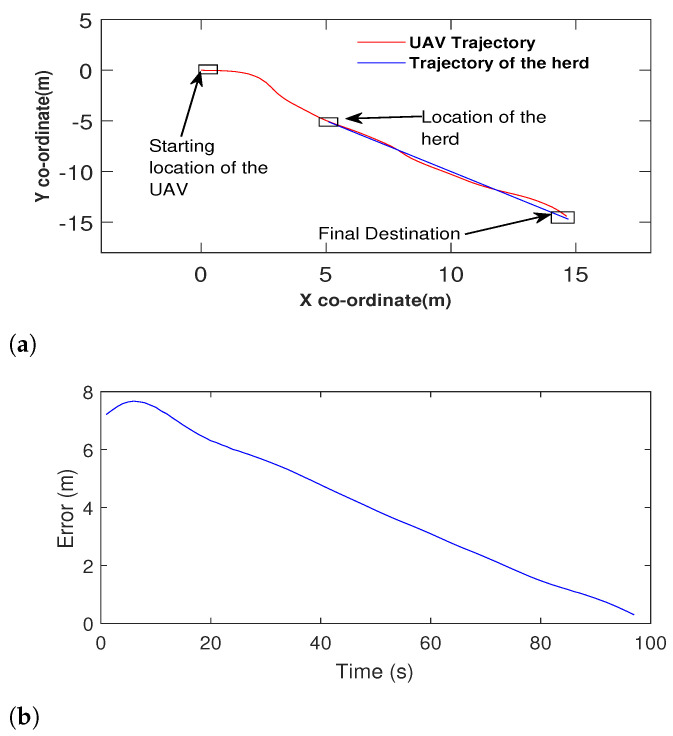
(**a**) Drone navigating towards a herd moving in a linear path. (**b**) The distance between the drone and the herd expressed as an error over time.

**Figure 14:**
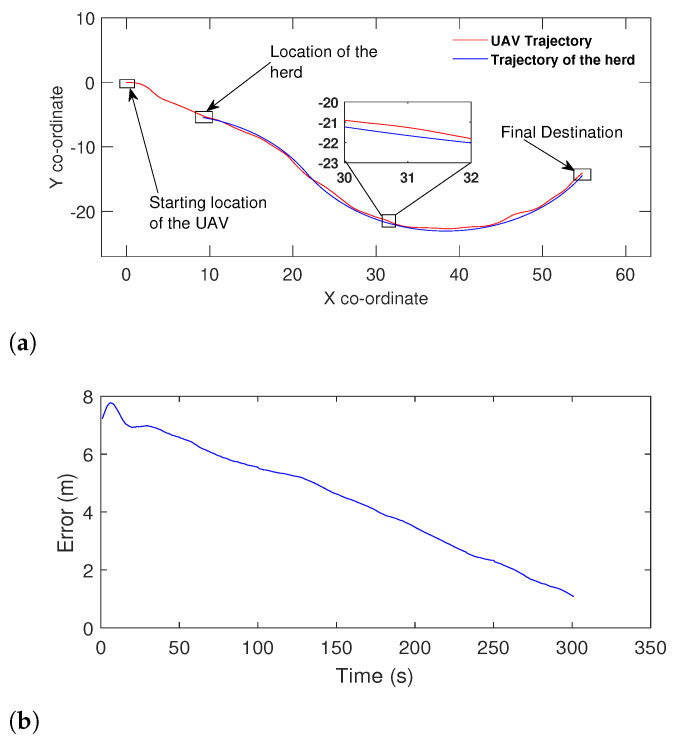
(**a**) Drone navigating towards a herd moving in a curved path. (**b**) Error/distance between the herd and the drone over time.

Then the drone followed a herd from a particular location to a destination, Figure 13a and Figure 14a show the results obtained. The speed of the drone has made close to that of the herd in order to address the drone’s circulation issue, which was discussed in the simulations. The experiments with a dynamic target (moving herd) were conducted in an identical manner to the simulations. In Figure 13a the control Equation (4) has made the drone to gradually reduce the heading and navigate in the direction of the herd. However, similar to the static scenario in Figure 12, the wind has been a significant factor and has caused a minor drift from the herd’s path. The drone in Figure 14a has gradually increased the heading and moved towards the herd. Around (9, −6), the drone has reached the target (herd), or in other words, the controller has reached the origin of the error space through the sliding surface. The main cause for the errors in range between targets and drones in Figure 13a and Figure 14a are the disturbances from the wind. The wind resistance has the ability to make a significant impact on the speed of the drone. However, the results presented in Figure 13a and Figure 14a prove that the algorithm performs sufficiently and robustly in the presence of disturbances.

## 6. Conclusions

This paper presented a novel methodology that can be implemented on an aerial drone for following a herd of cattle. The proposed method uses range data, i.e., the distance between the herd of cattle and the drone, delivered by simple inexpensive sensors without requiring any directional information. The navigation algorithm guarantees that an autonomous aerial drone approaches and follows a herd of cattle using only range data. The robustness of the controller ensures guaranteed interception of the herd of cattle regardless of the system dynamics involved. Generally, range measurements are robust and resilient against signal noise. Moreover, the algorithm does not need the range data to be accurate, rather it only relies on the rate of change of range data, which substantially improves robustness. The theory behind the proposed navigation algorithm was presented in detail, and the simulations and experimental results demonstrated the effectiveness of the proposed method. Furthermore, the proposed algorithm has outperformed other work when tested under noise. An interesting direction for future research is to extend the proposed approach to a network of aerial drones, see, e.g., [56], combining the autonomous navigation approach of the current paper with various methods of control of networked systems, see, e.g., [57,58,59]. Another interesting direction for future research is combining the developed navigation algorithm with advanced robust control and estimation techniques (see, e.g., [60,61]) to handle large uncertainties and non-linearities that are typical for dynamic models of drone’s and herd’s motion.

## Data Availability

Not applicable.
